# Prevalence and its associated risk factors of intestinal parasitic infections among Yadot primary school children of South Eastern Ethiopia: a cross-sectional study

**DOI:** 10.1186/1756-0500-7-848

**Published:** 2014-11-26

**Authors:** Begna Tulu, Solomon Taye, Eden Amsalu

**Affiliations:** Microbiology, Immunology and Parasitology Department, College of Medicine and Health Sciences, Bahir Dar University, P. O. Box 79, Bahir Dar, Ethiopia; Microbiology, Immunology and Parasitology Department, College of Medicine and Health Sciences, Madawalabu University, P. O. Box 302, Bale Robe, Ethiopia; Nursing Department, College of Medicine and Health Sciences, Bahir Dar University, P. O. Box 79, Bahir Dar, Ethiopia

**Keywords:** Prevalence, Intestinal parasites, Yadot primary school children, Risk factors

## Abstract

**Background:**

Intestinal parasitic infections are posing significant morbidity worldwide. In Ethiopia, due to poor socio-economic status, intestinal parasitic infections are highly prevalent. The main aim of this study was to determine the prevalence of intestinal parasites and its associated risk factors among Yadot primary school children which is found in South-Eastern part of Ethiopia, in the district called Delo-Mena.

**Methods:**

Institution based cross-sectional study was employed from March to April 2013. In this study, a total of 340 students were selected using simple random sampling, and data on socio-demographic characteristics and factors associated with the prevalence of intestinal parasites as well as stool samples were collected and processed accordingly. Statistical analysis was done using SPSS version 16, and binary and multivariate logistic regression analysis were conducted to measure the strength of association between dependent and independent variables.

**Results:**

The overall prevalence of intestinal parasites was 26.2%. Poly-parasitism was detected in 6.2% of the students. Consistently, students who were infected with single, double, triple and quadruple parasites were 20%, 4.7%, 1.2% and 0.3% respectively. In line with this, the most prevalent parasites were *Schistosoma mansoni* 12.6%, followed by *Entamoeba histolytica/dispar* 5%, *Ascaris lumbricoides* 4.7%, and *Hymenolepis nana* 4.4%. Regarding the risk factors for the infections, not knowing why they wash their hands before meal [(AOR = 0.20, 95% CI = 0.10-0.40), p < 0.001], water contact activities [(AOR = 2.28, 95% CI = 1.19-4.34), p = 0.012], not wearing protective shoe [(AOR = 0.27, 95% CI = 0.15-0.51), p < 0.001] were factors significantly associated with intestinal parasitic infections.

**Conclusion:**

Intestinal parasitic infections were found to be highly prevalent among Yadot primary school children. Hence, health education, improving sanitation, provision of safe drinking water, increasing latrine use, snail control and deworming to the students are crucial.

## Background

Intestinal Parasitic Infections (IPIs) are among the most prevalent of human parasitic infections worldwide, causing significant morbidity and mortality [1, 2]. More than 3.5 billion people are affected, and that 450 million are ill as a result of these infections, the majorities are being children [3–5]. Up to 250 million people are estimated to be infected with at least one or more species of intestinal nematodes in Sub-Saharan African countries. School age children and pregnant women are among the high-risk groups for intestinal parasitic infections [6–8]. These infections are also known as serious public health problems because the complications such as iron deficiency anaemia, growth retardation in children and other physical and mental health problems with serious consequences may occur [4–9].

In Ethiopia, IPIs are often associated with the existing low socio-economic status, poor personal and environmental sanitation, overcrowding, inadequacy and lack of safe water supply, tropical climate and low altitude [2–5]. Studies on the prevalence and distribution of IPIs in the country indicated that the type of parasites varies from place to place depending on the altitude. These studies also highlighted that *Ascaris lumbricoides* is the most prevalent intestinal parasite, followed by *Trichuris trichiura*, and hookworm [2–4, 9]. In the present study area, there was no published data regarding the prevalence and distribution of intestinal parasites. Thus, the objective of this study was to determine the prevalence of intestinal parasites and to identify associated risk factors among the Yadot primary school children in Delo-Mena district.

## Methods

This cross-sectional study was conducted from March to April 2013 in Yadot primary school, in Delo-Mena district, Bale Zone, Ethiopia. The district is situated towards South Eastern Ethiopia around 600 kms from the capital city, Addis Ababa. Its altitude is less than 1,500 meters above sea level. The area is also known by its production of cereals, chickpeas and haricot beans as important crops, and coffee is the main cash crop in the area [10]. Yadot primary school is found in the center of Delo-Mena town where almost all the students are urban residents.

The sample size was determined using the single proportion population formula, taking the prevalence rate of IPIs at Babile primary school, Eastern Hararghe Zone of Ethiopia (P = 27. 2%) [11]. For the calculation a 95% confidence interval (Zα/2), and 5% margin of error (d) was used. The calculated sample size was 351 including the fifteen percent (15%) non respondent rate. So, out of 1,944 students in Yadot primary school, 340 students were selected proportionally using simple random sampling. The school was stratified to grades, and the number of study participants were allocated proportionally to each grades based on their number of sections and number of students. However, students who were on active treatment were excluded from the study.

### Sample collection and microscopic examination

Data on socio-demographic, environmental and behavioral factors, and associated risk factors were collected by diploma nurses who were selected and trained for the purpose of this study. To ensure reliable information, the participant children were interviewed using their mother tongues. For those students who were unable to respond to questions properly, their guardians were contacted through the school principal. During the conversation, students were inspected by interviewers whether the students’ finger nails were trimmed or not, their general hygienic situation, dirty materials in their right hands, and their foot wears.

Students who were selected for the study were instructed on how to collect stool samples and provided the labelled clean plastic container, toilet tissue paper, and pieces of applicator sticks. As soon as the stool samples arrived, all samples were checked for their label, quantity, time, and procedure of collection.

### Laboratory procedure

Direct stool examinations were done by laboratory technologists using direct saline techniques within less than 20 minutes of stool sample collection. After the completion of direct stool examination, a portion of each sample was emulsified in a 10% formalin solution and transported to the Madawalabu University Biomedical Laboratory, where formal-ether concentration techniques were performed to increase the chance of detecting parasites. Iodine staining was also used to identify the cyst of *E. histolytica/dispar* from commensal *Entamoeba coli*.

### Quality control

To ensure the quality of the results, data collectors were trained for one day on how to conduct an interview, and how to collect stool samples. Completeness of the questionnaires was checked soon after the collection. Laboratory examinations were performed by experienced medical laboratory professionals who were working in the public hospitals. Stool samples were randomly selected for quality control purpose and examined by experienced laboratory technologists who were blinded to test.

### Data analysis procedures

Data entry and analysis were done using SPSS version 16 computer software. The baseline characteristics of the study population were summarized using medians and ranges for continuous variables; simultaneously, proportions and frequencies were used for categorical variables. Chi square test was calculated for the trends, and internal comparisons were made using logistic regression to determine the independent effect of the variables by calculating the strength of the association between infection and risk factors using odds ratio (OR) with 95% confidence interval (CI). Crude and adjusted OR were computed using bivariate and multivariable logistic regression analysis respectively. A p value less than 0.05 was considered as statistically significant.

### Ethical consideration

The study was approved by the Research Ethics and Review Committee of Madwalabu University. Participation was fully voluntary and informed written consent was obtained from each study subjects. For those children whose ages were under fifteen or unable to understand the purpose of the study, written consents were obtained from their family through school directors. Appropriate treatments were given to those students who were positive for IPIs by local nurses.

## Result

### Socio-demographic characteristics

A total of 340 students were considered in the analysis of this study with the non-response rate of 3.13%. Almost all 339 (99.7%) of the students reported that they are urban residents, and the mean age of the respondents was 11.08 years (satd. deviation ±2.6), and male to female ratio of 1.02:1.

Most of the students mothers’ educational status was classified under the level unable to read and write, that is, 148 (43.5%), followed by primary school (grade 1-8) 143 (42.1%), and high school and above (grade 9 and above) 49 (14.4%). Regarding parents’ occupation, merchant accounts 141 (41.5%), followed by employee 95 (27.9%), farmer 78 (22.9%), and unemployed 26 (7.6%). Sex (p < 0.001), family size (0.044), and mothers’ educational status (p < 0.001) were found to be significantly associated with the IPIs among the socio demographic characteristics of the students (Table 1).

**Table 1:** **Socio demographic characteristics of the students from Yadot primary school, Delo-Mena district, South Eastern Ethiopia, 2013**

Characteristics		Total (%)	No. of + ve for IPIs* (%)	P-value**
Sex	Male	172 (50.6)	61 (35.5)	<0.001
	Female	168 (49.4)	28 (16.7)	
Age in years	6-9	101 (29.7)	23 (22.8)	0.603
	10-14	203 (59.7)	57 (28.1)	
	≥15	36 (10.6)	9(25.0)	
Grade level	Grade 1-4	216 (63.5)	59 (27.3)	0.529
	Grade 5-8	124 (36.5)	30 (24.2)	
Family size	≤5	161 (47.4)	34 (21.1)	0.044
	≥6	179 (52.6)	55 (30.7)	
Number of rooms	≤3	275 (80.9)	77 (28.0)	0.116
	≥4	65 (19.1)	9 (18.5)	
Mothers’ educational status	Unable to read and write	148 (43.5)	56 (37.8)	<0.001
	Primary school	143 (42.1)	27 (18.9)	
	High school& above	49 (14.4)	6 (14.0)	
Parents’ occupation	Merchant	141 (41.5)	36 (25.5)	0.230
	Employee	95 (27.9)	25(26.3)	
	Farmer	78 (22.9)	25 (32.1)	
	Unemployed	26 (7.6)	3 (11.5)	

*IPIs = Intestinal Parasitic Infections** p-value of χ ^*2*^ *test for the trends.*

### Prevalence of IPIs

Based on microscopic stool sample examinations, ten species of intestinal parasites were identified with an overall prevalence of 26.2%. In other words, Poly-parasitism was detected in 6.2% of the students. In addition, double infection was observed in 4.7% of the students, and triple infection was seen in 1.2% of the participants. Moreover, a quadruple infection was detected in 0.3% of the students (Table 2).

**Table 2:** **Proportion of cases with mono-parasitism and poly-parasitism of IPIs among Yadot primary school children, Delo-Mena district, South Eastern Ethiopia, 2013**

Type of infection	No species	Species associated	Cases (%)
Mono-parasitism	1 species (n = 68)	*S. mansoni*	27 (7.9)
		*E. histolytica/dispar*	11 (3.2)
		*H. nana*	10 (2.9)
		*A. lumbricoides*	8 (2.4)
		*S. stercolaris*	5 (1.5)
		*G. lamblia*	4 (1.2)
		*T. trichiura*	2 (0.6)
		*E. vermicularis*	1 (0.3)
		Total mono-parisitism	68 (20)
Poly-parasitism	2 species (n = 16)	*A. lumbricoides and S. mansoni*	4 (1.2)
		*E. histolytica/dispar and G. lamblia*	3 (0.9)
		*G. lamblia and S. mansoni*	2 (0.6)
		*E. histolytica/dispar and H. nana*	1 (0.3)
		*E. vermicularis and S. mansoni*	1 (0.3)
		Hookworm and *S. mansoni*	1 (0.3)
		*H. nana* and *T. trichiura*	1 (0.3)
		*H. nana* and *S. mansoni*	1 (0.3)
		*A. lumbricoides* and *H. nana*	1 (0.3)
		*E. histolytica/dispar* and *S. mansoni*	1 (0.3)
		Sub-total	16 (4.7)
	3 species (n = 4)	*A. lumbricoides, T. trichiura* and *S. mansoni*	2 (0.6)
		Hookworm, Taenia spp and *S. mansoni*	1 (0.3)
		*H. nana, T. trichiura* and *S. mansoni*	1 (0.3)
		Sub-total	4 (1.2)
	4 species (n = 1)	*A. lumbricoides, T. trichiura, S. mansoni and E. histolytica/dispar*	1 (0.3)
		Total poly-parasitism	21 (6.2)

As can be observed from Table 3 below, the most prevalent parasites were *S. mansoni* 12.6%, followed by *E. histolytica/dispar* 5%, *A. lumbricoides* 4.7%, and *H. nana* 4.4%. When we see the overall prevalence of intestinal parasites, it was high among males (46.2%) compared to females which was (22.7%). Similarly, *S. mansoni* was significantly higher among male students (17.4%) compared to females (7.7%). In the same vain, the difference in the prevalence of *E. histolytica/dispar* among sexes was also found statistically significant, and the data revealed higher proportion among males compared to females with 7.6% and 2.4% respectively (Table 3).

**Table 3:** **Type of intestinal parasites identified among Yadot primary school children, Delo-Mena district, South Eastern Ethiopia, 2013**

Type of intestinal Parasite	Total positive n (%)	Male	Female	P value
*S. mansoni*	43 (12.6)	30 (17.4)	13 (7.7)	0.007
*E. histolytica/dispar*	17 (5.0)	13 (7.6)	4 (2.4)	0.029
*A. lumbricoides*	16 (4.7)	8 (4.7)	8 (4.8)	0.962
*H. nana*	15 (4.4)	12 (7.0)	3 (1.8)	0.020
*G. lamblia*	8 (2.4)	7 (4.1)	1 (0.6)	0.035
*T. trichiura*	7 (2.1)	3 (1.7)	4 (2.4)	0.679
*S. stercolaris*	5 (1.5)	2 (1.2)	3 (1.8)	0.633
*E. vermicularis*	2 (0.6)	2 (1.2)	0	0.161
Hookworm	2 (0.6)	1 (0.6)	1 (0.6)	0.987
Taenia spp	1 (0.3)	0	1 (0.6)	0.311

### Factors associated with IPIs

A close look at the findings on Table 4 below on the risk factors associated with the IPIs show that knowledge about hand washing before meal (p < 0.001), eat raw or undercooked or unwashed vegetables (p = 0.002), habit of latrine usage (p = 0.001), types of latrine used (p = 0.014), habit of finger nail trimming regularly (p = 0.011), water contact activities (p = 0.007), and not wearing protective shoe (p < 0.001) were found to be significantly associated with IPIs. However, other factors like habit of eating raw or under cooked meat, availability of latrine at home, availability of dirty materials in the right hand fingers, shoe wearing habit, and personal hygiene were not significantly associated with the prevalence of IPIs and the p values were >0.05 (Table 4).

**Table 4:** **Bivariate analysis of factors associated with IPIs among students from Yadot primary school, Delo-Mena district, South Eastern Ethiopia, 2013**

Risk factors		Total (%)	Positive No. (%)	Crude OR (95% CI)	P- value
Habit of hand washing before meal	Always	320 (94.1)	82 (25.6)	1.56[0.60-4.05]	0.358
	Sometimes	20 (5.9)	7 (35.0)	1	
Reason of washing hands before meal	Known	279 (82.1)	55 (19.7)	5.13[2.86-9.21]	<0.001
	Not known	61 (17.9)	34 (55.7)	1	
Eat raw or undercooked or unwashed vegetables	Yes	162 (47.6)	55 (34.0)	0.46[0.28-0.754]	0.002
	No	178 (52.4)	34 (19.1)	1	
Latrine availability at home	Yes	322 (94.7)	84 (26.1)	1.09[0.38-3.15]	0.874
	No	18 (5.3)	5 (27.8)	1	
Habit of latrine usage	Always	272 (80.0)	60 (22.1)	5.10[2.08-12.52]	0.001
	Sometimes	46 (13.5)	16 (34.8)	2.71[0.95-7.69]	<0.001
	Not at all	22 (6.5)	13 (59.1)	1	
Types of latrine used	Individual	288 (84.7)	68 (23.6)	2.42[0.78-7.54]	0.014
	Public	29 (8.5)	9 (31.0)	3.53[1.49-8.36]	0.004
	Open field	23 (6.8)	12 (52.2)	1	
Habit of finger nail trimming regularly	Yes	214 (62.8)	46 (21.5)	1.89[1.16-3.09]	0.011
	No	126 (37.1)	43 (34.1)	1	
Water contact activities	Yes	215 (63.2)	67 (31.2)	0.47[0.27-0.81]	0.007
	No	125 (36.8)	22 (17.6)	1	
Dirty materials in the right hand fingers	Present	167 (49.1)	43 (25.7)	1.04[0.64-1.69]	0.860
	Absent	173 (50.9)	46 (26.6)	1	
Protective shoe	Present	265 (77.9)	49 (18.5)	5.04[2.91-8.73]	<0.001
	Absent	75 (22.1)	40 (53.3)	1	
Personal hygiene	Good	233 (68.5)	59 (25.3)	1.15[0.68-1.92]	0.597
	Poor	107 (31.5)	30 (28.0)	1	

When we scrutinize the quantitative data on Table 5 below after adjusting for those variables which were statistically significant with *x*^*2*^test and in binary logistic analysis (p < 0.20), factors like not knowing why they wash their hands before meal (p < 0.001), water contact activities (p = 0.012), and not wearing a protective shoe (p < 0.001) were significantly associated with IPIs.

**Table 5:** **Adjusted OR of risk factors with IPIs among the students from Yadot primary school, Delo-Mena district, South Eastern Ethiopia, 2013**

Risk factors		Positive no. (%) for IP	Crude OR (95% CI)	P value	Adjusted OR	P value
Sex	Male	61 (35.5)	0.36[0.22-0.61]	<0.001	-	-
	Female	28 (16.7)	1		-	
Family size	≤5	34 (21.1)	1.66[1.01-2.72]	0.045	-	
	≥6	55 (30.7)	1		-	-
Number of rooms	≤3	77 (28.0)	0.58[0.29-1.15]	0.119	-	
	≥4	9 (18.5)	1		-	-
Mothers’ educational status	Unable to read and write	59 (37.8)	0.27[0.11-0.67]	<0.001	-	-
	Primary school	27 (18.9)	0.69[0.27-1.82]	0.005	-	
	Secondary school & above	6 (14.0)	1		-	
Reason of washing hands before meal	Known	55 (19.7)	5.13[2.86-9.21]	<0.001	4.93[2.47-9.85]	<0.001
	Not known	34 (55.7)	1		1	
Eat raw or undercooked or unwashed vegetables	Yes	55 (34.0)	0.46[0.28-0.754]	0.002	-	-
	No	34 (19.1)	1		-	-
Habit of latrine usage	Always	60 (22.1)	5.10[2.08-12.52]	0.001	-	
	Sometimes	16 (34.8)	2.71[0.95-7.69]	<0.001	-	
	Not at all	13 (59.1)	1		-	
Habit of finger nail trimming regularly	Yes	43 (48.3)	1.89[1.16-3.09]	0.011	-	-
	No	46 (51.2)	1		-	-
Water contact activities	Yes	67 (75.3)	0.47[0.27-0.81]	0.007	0.44[0.23-0.83]	0.012
	No	22 (24.7)	1		1	
Protective shoe	Present	40 (44.9)	5.04[2.91-8.73]	<0.001	3.66[1.96-6.86]	<0.001
	Absent	49 (55.1)	1		1	

Consistent with the above facts, students who did not know the reason why they wash their hands before meals were found to be five times (AOR = 4.93, 95% CI = 2.47-9.85) more likely to be infected with IPIs than other students who washed their hands. Similarly, students who did not wear protective shoe were also found to be almost four times (AOR = 3.66, 95% CI = 1.96-6.86) more likely to be to be infected with IPIs compared to those who wear protective shoe. In addition, students who claimed they had no frequent contact with water during swimming and irrigation activities were found to be fifty six percent protected (AOR = 0.44, 95% CI = 0.23-0.83) to be infected by IPIs compared to those who were unable to do so (Table 5).

## Discussion

This study assessed the prevalence of IPIs, and its associated risk factors in Yadot primary school of Delo-Mena district, Bale zone, South Eastern Ethiopia. The overall prevalence of intestinal parasites was found to be 26.2% which is lower than similar previous studies conducted in different parts of Ethiopia, for instance in Langano (83.8%) [2], Delgi (79.8%) [3], and Jimma (83%,47.1%) [4, 9] and in other countries like Pakistan (54.4%) [12], Nigeria (35.98%, 51.54%) [1] and India (42.8%) [13]. In contrast, the present study showed very much higher prevalence of IPIs compared to the study conducted in Italy (13.24%, 10%) [14]. These differences in prevalence could be due to the place and living standard of study subjects, and/or due to a reflection of the local endemicity and geographic condition of the study area.

*S. mansoni* (12.6%) was found to be the highest prevalent parasite in this study, which is significantly higher as compared to the study done in other part of Ethiopia, Babile (4.3%) [11]. The high prevalence of *S. mansoni* in this study might be associated to the river Yadot which pass through the Delo-Mena town, and the behavioural factors of the students (such as frequent contact, drinking and swimming in the river). On the contrary, the prevalence of *S. mansoni* in this study was much lower than the previous studies conducted in other areas of Ethiopia, like South Gonder (14.6%) [15], Langano (21.2%) [4], and Delgi (15.9%) [3]. Since we could not get published paper regarding the topic in concern, it is highly recommended to undertake further studies to identify the transmission foci that will enable to design methods of prevention and control in a systematic way.

The second most prevalent intestinal parasite identified in this study was *E. histolytica/dispar* (5.0%) which was very much lower than a study conducted in Ethiopia, Delgi (27.3%) [3] and in other country, Tajikistan (25.9%) [6]. *A. lumbricoides* (4.8%) and *H. nana* (4.4%) were the third and fourth most prevalent parasites identified in this study respectively. Especially, the result of *A. lumbricoides* in this study was significantly lower than other studies conducted in Ethiopia specifically in North Gonder, Delgi (48%) and Wondo Genet (84.3%) [3, 16], and in other countries, such as Guinea (18.9%) and Nigeria (53.4%) [17, 18]. However, it is almost similar with the study conducted in Ethiopia around lake Langano (6.2%) [2] and in Pakistan (4%) [12]. In line with this, even though environmental sanitation and personal hygiene of study subjects probably play an important role for the higher prevalence rate of Ascariasis, there was lowest prevalence of the parasite identified in this study area, and this needs further explanations.

The magnitudes of other parasites identified in this study were also comparable with the findings of other study areas. In localities where numerous kinds of intestinal parasites found, multiple infections were frequently encountered. In this study, double (6.2%), triple (1.2%) and quadruple (0.3%) infection were observed. The most frequent mixed infections were *A. lumbricoids* and *S. mansoni*. Whereas, studies from other areas showed *A. lumbricoids, T. trichiura* and Hookworm were the most common mixed infections [17–19]. This is probably due to environmental conditions and methods of transmission that make favorable for the two parasites to live together, or the occurrence of high prevalence of the two parasites in the study area.

In this study, the possible association of IPIs with potential risk factors among school children was also assessed. Several recent studies have identified ranges of environmental, behavioral and social risk factors associated with intestinal parasite infections. *S. mansoni, E. histolytica/dispar, H. nana,* and *G. lamblia* seriously affected males than females, and the associations were statistically significant (p < 0.05). The reason for the associations are not clear, but as to schistosomiasis, males may have higher exposure to river water than females [4]. Though it is not significantly associated with IPIs in this study, low educational status of children’s mother is known to be among the significant factors in recent studies conducted in Ethiopia and other countries [3, 5, 20]. This can be explained as the parents of children at high level of educational status can provide better sanitation condition for their children than those parents with low educational level.

Consistently, in this study, significantly higher prevalence of IPIs was detected among students who did not know why they wash their hands before meal compared to those who knew why they wash their hands before meal. Similarly, the other factors that exposed children to IPIs identified in this study were water contact activities (like playing, swimming, fishing and irrigation), and not wearing protective shoe. These findings are consistent with other studies conducted in Ethiopia [3, 4, 11]. This is probably due to low knowledge of children about the feco-oral transmission of intestinal parasite, and frequent contact with contaminated water sources may serve as the source of infections in many cases.

Some of the limitations of this study include the microscopy techniques which may underestimate the prevalence of intestinal parasites in the study population. Other techniques like molecular assays could best estimate the prevalence of intestinal parasites among the study subjects. However, due to financial constraints, we were unable to perform these advanced tests. Selection bias may also arise from convenience sampling.

## Conclusion

In conclusion, intestinal parasitic infection is highly prevalent among school children in Delo-Mena district. Among these infections, *S. mansoni* is the most prevalent parasite followed by *E. histolytica/dispar*, *A. lumbricoides*, and *H. nana*. Factors like not knowing the reason why they wash their hands before meal, water contact activities, and not wearing protective shoe were significantly associated with intestinal parasite infections in the district. Hence, health education, improving sanitation, provision of safe drinking water, increasing latrine use, snail control and deworming to the students are crucial. Also further studies should be conducted to identify the transmission foci of the identified intestinal parasites.
